# Plant Clonal Integration Mediates the Horizontal Redistribution of Soil Resources, Benefiting Neighboring Plants

**DOI:** 10.3389/fpls.2016.00077

**Published:** 2016-02-05

**Authors:** Xue-Hua Ye, Ya-Lin Zhang, Zhi-Lan Liu, Shu-Qin Gao, Yao-Bin Song, Feng-Hong Liu, Ming Dong

**Affiliations:** ^1^State Key Laboratory of Vegetation and Environmental Change, Institute of Botany, Chinese Academy of SciencesBeijing, China; ^2^University of Chinese Academy of SciencesBeijing, China; ^3^Key Laboratory of Hangzhou City for Ecosystem Protection and Restoration, Hangzhou Normal UniversityHangzhou, China; ^4^National Science Library, Chinese Academy of SciencesBeijing, China

**Keywords:** clonal integration, deuterium, environmental heterogeneity, [^15^N], hydraulic redistribution, *Potentilla anserina*, stable isotope

## Abstract

Resources such as water taken up by plants can be released into soils through hydraulic redistribution and can also be translocated by clonal integration within a plant clonal network. We hypothesized that the resources from one (donor) microsite could be translocated within a clonal network, released into different (recipient) microsites and subsequently used by neighbor plants in the recipient microsite. To test these hypotheses, we conducted two experiments in which connected and disconnected ramet pairs of *Potentilla anserina* were grown under both homogeneous and heterogeneous water regimes, with seedlings of *Artemisia ordosica* as neighbors. The isotopes [^15^N] and deuterium were used to trace the translocation of nitrogen and water, respectively, within the clonal network. The water and nitrogen taken up by *P. anserina* ramets in the donor microsite were translocated into the connected ramets in the recipient microsites. Most notably, portions of the translocated water and nitrogen were released into the recipient microsite and were used by the neighboring *A. ordosica*, which increased growth of the neighboring *A. ordosica* significantly. Therefore, our hypotheses were supported, and plant clonal integration mediated the horizontal hydraulic redistribution of resources, thus benefiting neighboring plants. Such a plant clonal integration-mediated resource redistribution in horizontal space may have substantial effects on the interspecific relations and composition of the community and consequently on ecosystem processes.

## Introduction

The hydraulic redistribution (Burgess et al., 1998) of soil water have been observed to play important roles in many ecosystems, particularly in arid and semiarid ones (Nadezhdina et al., 2010; Neumann and Cardon, 2012; Hao et al., 2013). Though, theoretically speaking, such a hydraulic redistribution can be vertical and/or horizontal, vertical hydraulic redistribution of soil water (or hydraulic lift, Richards and Caldwell, 1987) has received much more attention (Caldwell et al., 1998; Horton and Hart, 1998; Brooks et al., 2006). In the vertical hydraulic redistribution of water, a plant root system takes up water from the soil and translocates it upward before releasing it into an upper layer of the soil (Prieto et al., 2010; Robinson et al., 2012). This vertical hydraulic redistribution of water is a benefit not only to the donor plants (Querejeta et al., 2003) but also to the surrounding biota because it provides an alternative source of water (Ludwig et al., 2004; Brooks et al., 2006). The redistribution of water is also a mechanism through which nutrients in deeper soils are mobilized for uptake by plants (Wang et al., 2009).

Natural environments are often heterogeneous and consist of patches with contrasting levels of resources (García-Palacios et al., 2012). Many clonal plants, particularly stoloniferous or rhizomatous clonal plants, create a clonal network that consists of a large number of interconnected ramets, which may occupy patches that are widely spaced and differ in resource availability (Jońsdóttir and Watson, 1997; Charpentier et al., 2012; Li et al., 2012). A large body of evidence has demonstrated that ramets growing in high resource patches (donor ramets in donor microsites) translocate resources to the interconnected ramets growing in low resource patches (recipient ramets in recipient microsites), and this translocation occurs through horizontal structures such as rhizomes, stolons or roots (Atkinson and Else, 2012; Roiloa and Hutchings, 2013; Luo et al., 2014; Pinno and Wilson, 2014; Roiloa et al., 2014). Such clonal integration greatly improves the performance of the recipient ramets and often also that of the entire clone (Yu et al., 2004; Song et al., 2013).

Thus, soil resources such as water and nutrients are translocated from donor to recipient ramets via clonal integration (Alpert, 1999; Wang et al., 2014; Dong et al., 2015). For some clonal plants with a large clonal network, the distance covered can be large after multiple translocations from donor to recipient ramets. Additionally, the resources are released into the soil through the roots of the recipient ramets, driven hydraulically by a water potential gradient (McCully, 1995; Steudle and Frensch, 1996). Therefore, we hypothesized that soil resources, before being released into recipient microsites, are translocated for relatively long distances among microsites by the horizontal redistribution of soil resources through an integrated clonal network from donor to recipient ramets. Furthermore, we hypothesized that neighboring plants use the release of water and possibly nutrients from the clonal network to improve growth. Although horizontal redistribution of soil resources is vital to understand facilitative effect among plant individuals, especially in dryland ecosystems, so far no study has explicitly tested whether a horizontal redistribution of soil resources can be mediated by clonal integration and whether it can further impact the growth of neighbor plants.

To test these hypotheses, we conducted two greenhouse experiments with the stoloniferous, clonal plant *Potentilla anserina*. We grew ramets of *P. anserina* in a high-resource (donor) microsite that were connected to the ramets of *P. anserina* with a neighbor *Artemisia ordosica* seedling in a low-resource (recipient) microsite. The isotopes [^15^N] and deuterium ([D]) were used to trace and quantify the redistribution of nitrogen and water, respectively, from the donor microsite to the recipient microsite via ramets of *P. anserina* interconnected by stolons. We expected that the tracers added to the donor microsite would be detected in the recipient microsite and in the neighboring plant (*A. ordosica*) and that the growth of the neighboring plant would be improved by the horizontal hydraulic redistribution of water and nutrients mediated by clonal integration.

## Materials and Methods

### Plant Species

*Potentilla anserina* L. (Rosaceae) is a clonal, rosette-forming herb with sympodial, plagiotropic stems (stolons; Ma, 1989). Along a stolon, each node produces leaves and roots under moist conditions, forming a potentially independent ramet. In northern China, the stolons wither during autumn and winter, and the ramets become completely independent (Ma, 1989). All ramets of *P. anserina* used in this study were vegetatively propagated from a single clone collected on 10th August 2008 near the Ordos Sandland Ecological Station (OSES, 39°02′ N; 109°21′ E), which is located in the northeastern section of the Mu Us Sandland in Inner Mongolia, China. The *P. anserina* clone was cultivated in the greenhouse at OSES to obtain sufficient ramets of similar size and age for the experiments.

The semi-shrub *A. ordosica* Krasch (Asteraceae) is a dominant species in the Mu Us Sandland. The root system has abundant lateral roots, primarily distributed in the upper, dry sand layer (Li et al., 2010). In sandlands such as Mu Us, *P. anserina* inhabits the lowlands between dunes and also often spreads into the dunes that are dominated by *A. ordosica*.

### Experiments

#### Experiment I

**FIGURE 1:**
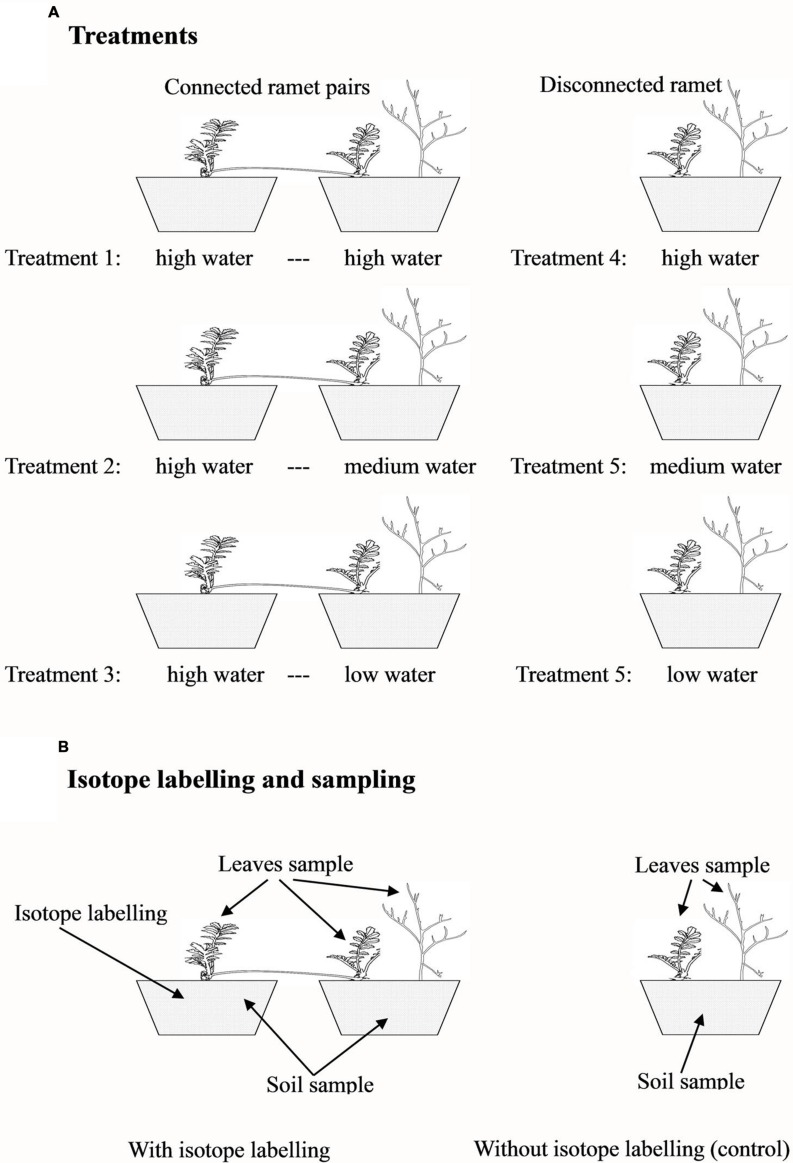
**Experimental design.** Three water treatments for connected ramet pairs or for disconnected, single ramets **(A)**; and the sampling design with or without isotope labeling **(B)**.

To test whether clonal integration mediated the redistribution of nutrients among microsites, the first experiment was conducted in 2009. We selected 27 pairs of connected ramets and 27 pairs of severed (disconnected) ramets (three leaves per ramet) of *P. anserina*. Each ramet pair was planted in a pair of cylindrical plastic containers (21 cm in diameter and 21 cm in height) filled with sand collected near the field station. Each severed, developmentally younger ramet was planted in a container of the same size, but all the severed, developmentally older ramets were discarded. Ten days after transplantation, the connected ramet pairs (connected treatment) were randomly assigned to one of three water treatments (**Figure 1A**): (1) both ramets of a ramet pair were watered with 400 ml every 3 days (high water regime); (2) one ramet was watered with 400 ml every 3 days, and the connected ramet was watered with 300 ml (medium water regime); and (3) one ramet was watered with 400 ml every 3 days, and the connected ramet was watered with 200 ml (low water regime). In this experiment, the developmentally older ramet of each pair was always assigned to the high water treatment (in the potential donor container), and the connected, developmentally younger ramet was randomly assigned to the high, medium, or low water regime and was also grown with one *A. ordosica* seedling (in the potential recipient container). The different water regimes were used to create different water potential gradients between the two interconnected ramets of each pair to facilitate nutrient translocation through clonal integration (de Kroon et al., 1998). The 27 disconnected, developmentally younger *P. anserina* ramets (severed treatment in which the stolon internode connecting the two ramets of a pair was cut midway with scissors) were randomly assigned to one of the three water treatments (**Figure 1A**): (4) high water treatment with *A. ordosica*, (5) medium water treatment with *A. ordosica*, and (6) low water treatment with *A. ordosica*. All seedlings of *A. ordosica* used in this experiment were approximately 2 cm tall. Nine replicates were used per treatment. The experiment was conducted for 2 months from 15th August 2009 to 15th October 2009. Three days before harvest, three replicates from the three connected treatments were randomly selected, and the older ramets in the high water treatment were labeled with [^15^N]. For labeling, 400 ml of NH_4_^15^NO_3_ solution (containing 0.066 g ^15^N) was added to the containers with the older ramets to trace the movement of N. All the other containers, including the rest donor containers, all the recipient and disconnected containers with different water treatments, received the identical amount of NH_4_NO_3_ but at different concentrations. After harvest, three of the replicates (including all three labeled and three disconnected, unlabeled replicates) were sampled to determine the values of δ^15^N in the plant leaves and soils (**Figure 1B**), and the other six replicates were used to measure plant biomass. After oven-drying at 80°C for at least 48 h, the shoot and root biomass was measured for the younger ramet of each pair and the neighbor *A. ordosica* and for each disconnected ramet of *P. anserina* and the neighbor *A. ordosica*.

#### Experiment II

To exclude the effect of nutrients and to verify the effect of water on plant growth, the second experiment was conducted in 2011 to test whether the redistribution of water mediated by clonal integration benefited neighboring plants. We selected 30 pairs of connected ramets and 30 pairs of severed (disconnected) ramets (three leaves per ramet) of *P. anserina*. Each ramet pair was planted into a pair of cylindrical plastic containers (21 cm in diameter and 21 cm in height) filled with sand, and each disconnected, developmentally younger ramet was planted in a similar container (all the severed, developmentally older ramets were discarded). The experimental design was identical to that for Experiment I, with the exception that all *A. ordosica* seedlings were 40 days-old (approximately 15 cm tall) and that D_2_O was used for labeling 3 days before harvest (**Figure 1A**). The experiment began on 31st August 2011 and ended on 30th September 2011. Ten replicates were used per treatment. Three days before harvest, three replicates in the connected treatments were randomly selected, and the older ramets in the high water treatment were labeled with D_2_O. For the labeling, a 300 ml solution of 1/400 D_2_O solution (containing 99.8% [D] in D_2_O) was added to the containers to trace the movement of water, whereas the other containers not used for labeling received 300 ml of double distilled water. Three replicates (including the three labeled and the three disconnected, unlabeled replicates) were sampled to determine the values of δD in the leaves and soils (**Figure 1B**), and the other seven replicates were used to measure plant biomass, as in Experiment I.

### Isotope Measurements

We measured the [^15^N] or [D] concentrations (1) in the soils and in the *P. anserina* leaves in the labeled (donor) containers; (2) in the soils, *P. anserina* leaves and *A. ordosica* leaves in the unlabeled, paired (recipient) containers (growing with the connected *P. anserina* ramets); and (3) in the soils, *P. anserina* leaves and *A. ordosica* leaves in the unlabeled, control containers (growing with the single, severed *P. anserina* ramets) using a MAT253 Isotope Ratio Mass Spectrometer (Thermo Fisher Scientific, Inc., USA) coupled with an Elemental Analyser (Flash EA1112 HT, Thermo Finnigan, USA). The abundance [^15^N] isotope ratio (δ^15^N, ‰) and the [D] isotope ratio (δD, ‰) were calculated as follows:

(1) $$\delta^{15}N\,\;or\,\;\delta D=\left(R_{sample}/R_{s\tan dard}-1\right)\times 1000$$

where R_sample_ and R_standard_ are the ^15^N/^14^N ratio or the D/^1^H ratio in a sample and the standard, respectively. The standards were international standard atmospheric air N_2_ for nitrogen and standard mean ocean water for deuterium.

We calculated the ratios of RN and RD from the δ^15^N and δD values in the soils, *P. anserina* leaves or *A. ordosica* leaves in the labeled (donor) containers or unlabeled, paired (recipient) containers and those in the unlabeled, control containers as follows:

(2) $$RN=\delta^{15}N_{Exp}/\delta^{15}N_{Nat}$$

(3) $$RD={\delta D}_{Exp}/{\delta D}_{Nat}$$

where δ^15^N^Exp^ is the δ^15^N in the soils, *P. anserina* leaves or *A. ordosica* leaves in the labeled, donor containers or unlabeled, recipient containers; δ^15^N^Nat^ is the δ^15^N in the soils, *P. anserina* leaves or *A. ordosica* leaves in the corresponding unlabeled, control containers; RN is the ratio of δ^15^N in experimental materials to that of the controls; δD^Exp^ is the δD in the soils, *P. anserina* leaves or *A. ordosica* leaves in the labeled, donor containers or unlabeled, recipient containers; δD^Nat^ is the δD in the soils, *P. anserina* leaves or *A. ordosica* leaves in the corresponding unlabeled, control containers; and RD is the ratio of δD in the experimental materials to that in the controls. When RN > 1 or RD > 1, [^15^N] or [D] was translocated into the soil and plant, and high values of RN or RD indicated that more [^15^N] or [D] was translocated.

### Data Analyses

We used one-sample *t*-tests to determine whether the RN and RD in soils and in plant leaves in each water treatment in the unlabeled, recipient containers were significantly larger than 1 (one-tailed tests). One-way ANOVAs were used to test the effects of water treatment (high, medium, or low water supply) on the RN and RD in soils and plant leaves. When a significant effect was detected, Tukey’s HSD tests were used for multiple comparisons. We used two-way ANOVAs to analyze the effects of clonal integration (stolon severing or not) and water treatments (high, medium, or low water supply) on the biomass of *P. anserina* and *A. ordosica*. The data for the RN and the RD were log-transformed to meet the assumption of normality and homogeneity of variance. All statistical analyses were performed using the SPSS17.0 statistical software package (SPSS, Chicago, IL, USA).

## Results

In our experiment, the δ^15^N in control soils, *P. anserine* leaves and *A. ordosica* leaves was 7.691 ± 0.23‰, 29.45 ± 1.14‰, and 3.278 ± 0.21‰, respectively; and δD was -39.91 ± 0.97‰, -1.80 ± 0.92‰, and -44.29 ± 2.43‰, respectively. The RN and the RD, the ratios of δ^15^N and δD in labeled materials to those in control materials, respectively, were used to show the effects of isotope labeling and translocation. Higher values of RN or RD indicated that more of the isotope was in the experimental materials than in the control materials. In the labeled containers, the RN and RD in soils and *P. anserina* leaves were much greater than 1 (Supplementary Figure S1), which indicated that the isotopes were successfully added to the soil and absorbed by the plant.

**FIGURE 2:**
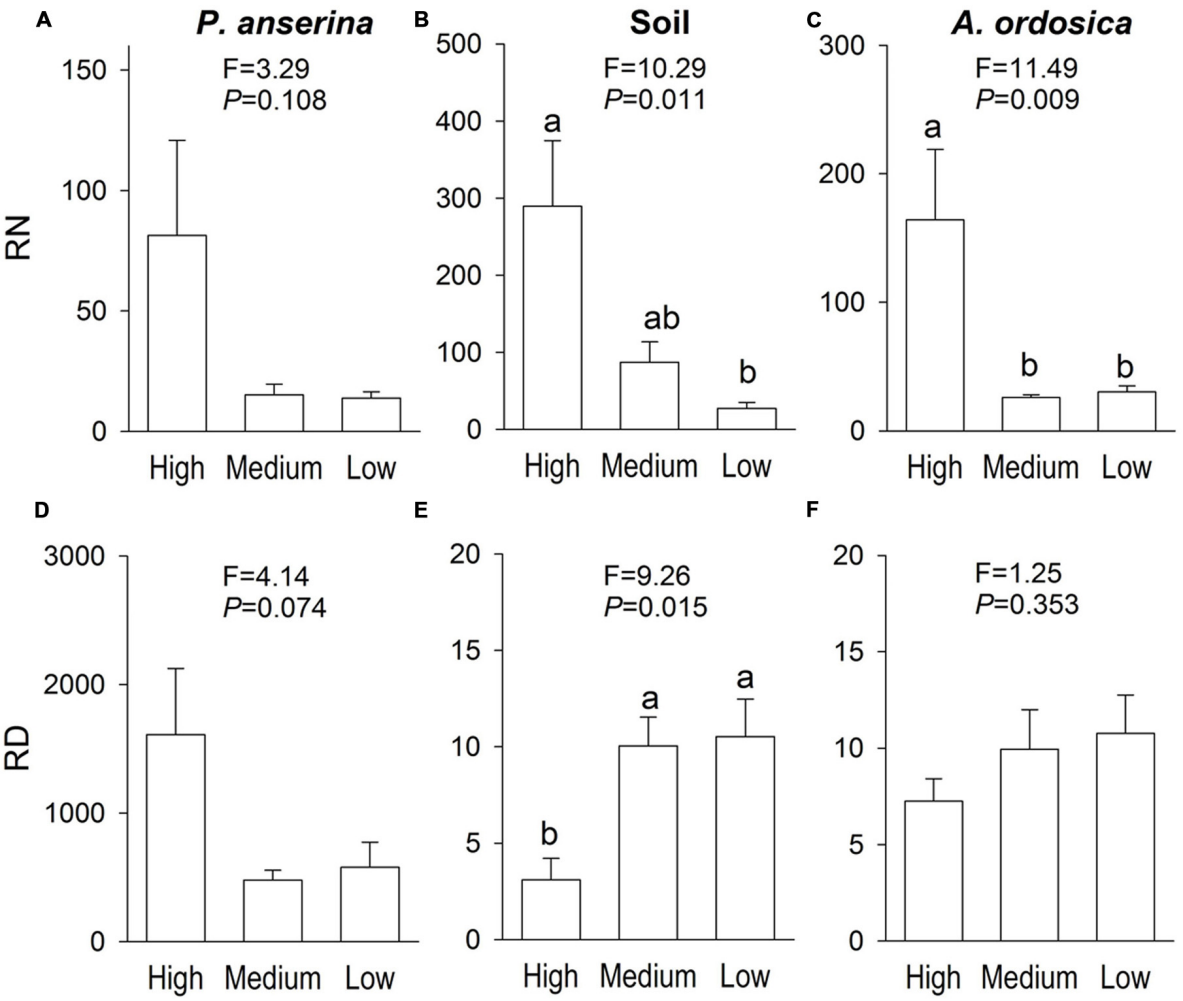
**The ratio of δ^15^N (RN, above, in Experiment I) and the ratio of δD (RD, below, in Experiment II) in *Potentilla anserina* leaves (A,D), soils (B,E), and *A. ordocia* leaves (C,F) in the unlabeled, recipient containers with the high, medium and low water treatments that were connected to the *P. anserina* ramets with the high water supply.** All means are significantly greater than 1 (one-sample *t*-tests). The *F* and *P*-values of the one-way ANOVA are also provided, and different letters indicate significant differences in means among treatments.

**FIGURE 3:**
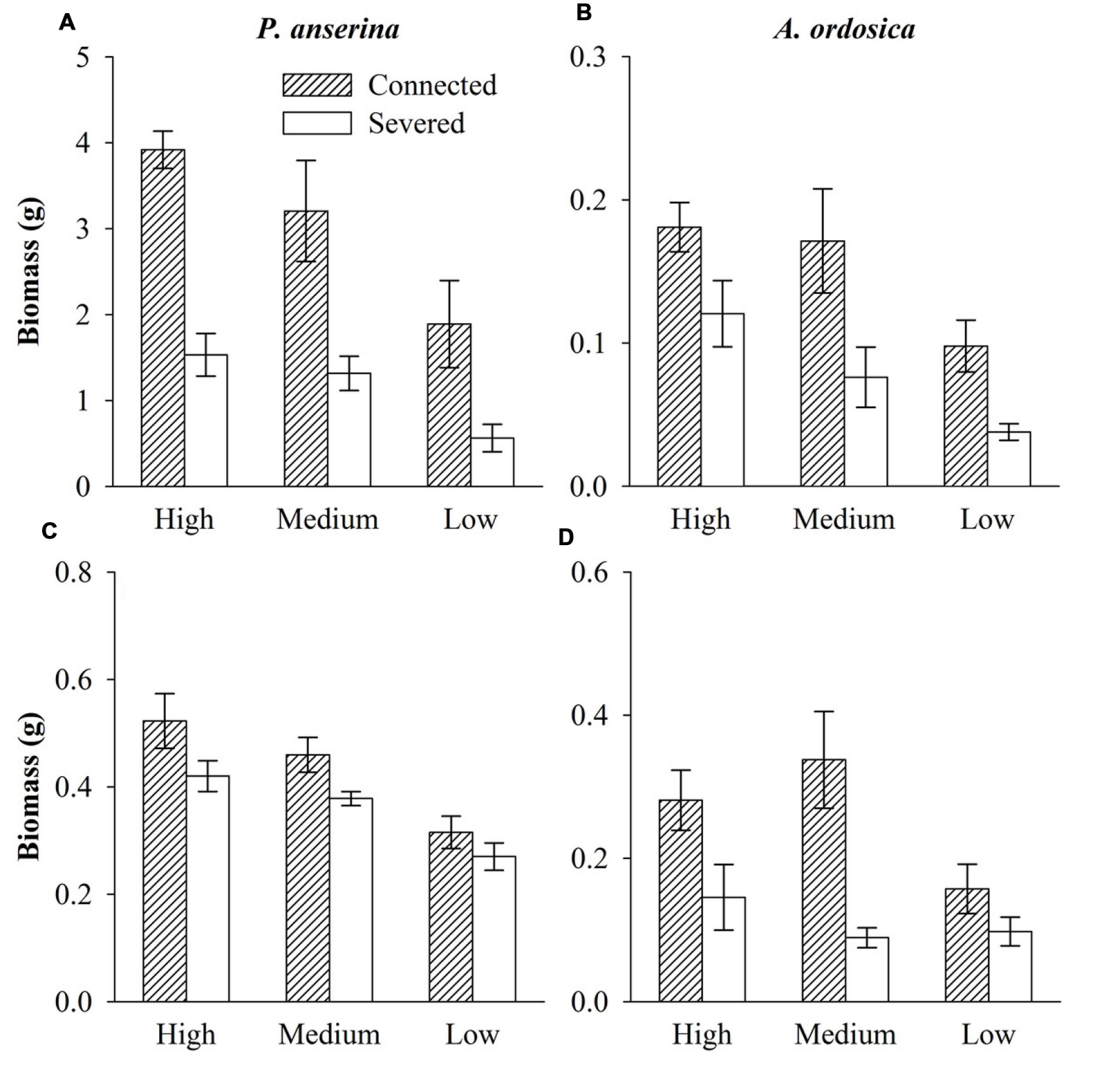
**Biomass of *P. anserina* (A,C) and *Artemisia ordosica* (B,D) in the unlabeled containers with the high, medium, and low water treatments that were connected with or severed from the *P. anserina* ramets grown in the high water treatment in Experiment I (A,B) and Experiment II (C,D)**.

In the unlabeled, recipient containers, both the RN and RD in *P. anserina* leaves were significantly greater than 1 (*P* < 0.05; **Figures 2A,D**), and the clonal integration significantly increased the biomass of *P. anserina* (**Table 1**; **Figures 2A,C**). These results demonstrated that [^15^N] and [D] were translocated from the donor ramet of *P. anserina* to the interconnected, recipient ramet, and through clonal integration, the growth of the recipient ramet increased. In the unlabeled, recipient containers, the RN and RD in soil and *A. ordosica* leaves were significantly larger than 1 (*P* < 0.05; **Figures 2B,C,E,F**), and through clonal integration, the biomass of *A. ordosica* increased significantly (**Table 1**; **Figures 3B,D**). Therefore, the increase in the growth of *A. ordosica* was probably attributable to the uptake of the translocated [^15^N] and [D] that were released by the recipient ramets into the soil.

**Table 1:** Effects of severing stolons and water treatment on biomass and shoot to root ratio of *Potentilla anserina* and *Artemisia ordosica* in **(A)** Experiment I and **(B)** Experiment II.

Effect		*P. anserina*				*A. ordosica*			
		Biomass		Root/shoot		Biomass		Root/shoot	
	DF	*F*	*P*	*F*	*P*	*F*	*P*	*F*	*P*
**(A)** Experiment I									
Severing (S)	1, 31	30.9	**<0.001**	26.2	**<0.001**	10.4	**0.003**	13.5	**0.001**
Water (W)	2, 31	5.2	**0.011**	0.6	0.556	4.1	**0.027**	15.6	**<0.001**
S × W	2, 31	0.6	0.574	6.8	**0.004**	0.4	0.662	3.8	**0.032**
**(B)** Experiment II									
Severing (S)	1, 29	5.3	**0.029**	1.3	0.272	15.1	**0.001**	0.2	0.652
Water (W)	2, 29	14.4	**<0.001**	2.4	0.106	2.3	0.118	1.7	0.200
S × W	2, 29	0.2	0.856	3.6	**0.040**	2.2	0.132	1.2	0.303

*Values in bold indicate significance at P < 0.05.*

The RN in soils and in *A. ordosica* leaves in the unlabeled, recipient containers was significantly higher in the high water supply treatments than in the low water supply treatment (**Figures 2B,C**); however, the RD in soils was significantly lower (**Figure 2E**), possibly because more of the water supply was diluted under high water treatment. The RN and RD in *P. anserina* leaves and the RD in *A. ordosica* leaves did not differ significantly among the three water treatments (**Figures 2A,D,F**). Furthermore, the effect of clonal integration on the growth of *P. anserina* and *A. ordosica* were also not dependent on the water regime (**Table 1**; **Figure 3**).

Severing stolon had significant effects on both root/shoot ratio of *P. anserina* and *A. ordosica*, and water treatment significantly affected root/shoot ratio of *A. ordosica* in Experiment I; while in Experiment II, neither severing stolon nor water treatment had significant effects on root/shoot ratio of *P. anserina* and *A. ordosica* (**Table 1**).

## Discussion

Our hypotheses were supported: via a clonal network, clonal integration mediated the horizontal redistribution of soil resources essential for plant growth and reproduction, between different microsites. Moreover, the redistribution of water and nitrogen increased the growth of neighboring plants.

The horizontal redistribution of soil resources is limited by the abilities to translocate resources and to release resources into the soil (Nadezhdina et al., 2010; Neumann and Cardon, 2012). Numerous studies have confirmed that resources are transported from one ramet to another via horizontal clonal organs, including stolons, rhizomes and roots, through clonal integration (Alpert, 1999; D’Hertefeldt and Jónsdóttir, 1999; van Kleunen et al., 2000; Roiloa and Hutchings, 2013). A large body of evidence has also indicated that plant roots release resources into the soil through hydraulic redistribution (Moreira et al., 2003; Leﬄer et al., 2005; Smart et al., 2005; Brooks et al., 2006). Our results show that water and nitrogen were translocated from a donor to a recipient ramet of *P. anserina* through clonal integration and then released by the roots of the recipient ramet into the soil, most likely through hydraulic redistribution. In a previous study based on deuterium labeling, (de Kroon et al. (1998) have also noted that soil water is translocated through a clonal network and then released into soil. However, with a model, Magyar et al. (2004) have shown that clonal integration may also mediate soil nutrient redistribution through decomposition; i.e., nutrients taken up by a donor ramet in the donor microsite are translocated to the recipient ramet in the recipient microsite and then released into the soil in the recipient microsite with the decomposition of the dead, recipient ramet. Although we could not fully eliminate the effect of root decomposition, we presumed that this effect released much less water and nitrogen than that of hydraulic redistribution because the duration of the experiment was short (not more than 2 months), and the isotopes were added only 3 days before harvest. Thus, the release of nutrients through the decomposition of roots was expected to be minimal. In cases in the field in which clonal plants form large, interconnected clonal networks, clonal integration mediates the long-distance translocation of resources (D’Hertefeldt and Jónsdóttir, 1999; Li et al., 2012). Therefore, for the species with large clonal networks that include a few dozen or 100s of ramets (Li et al., 2012; Song et al., 2013), we propose that the extent of resource redistribution in the field is substantial when resources are available and that the long-distance redistribution of resources may be possible through clonal integration. This area of research has promise for future study.

Most notably, the clonal integration in *P. anserina* significantly increased the biomass of *A. ordosica* grown as a neighbor to the recipient ramet of *P. anserina*. Thus, the neighbor *A. ordosica* must have absorbed a substantial amount of the water and nitrogen released by *P. anserina* into the soil. This interpretation was confirmed by isotope labeling; [^15^N] and [D] of the neighbor *A. ordosica* grown with an unlabeled, *P. anserina* ramet that was connected to a labeled ramet were much higher than those of *A. ordosica* grown with an unlabeled, disconnected ramet of *P. anserina*. Whereas previous studies show that resources released by a target plant through hydraulic lift can be used by neighboring plants and microbes (Burgess, 2011; Sekiya et al., 2011; Pang et al., 2013), we are the first to demonstrate that the resource redistribution mediated by clonal integration affects the growth of neighboring plants. Horizontal hydraulic redistribution of soil resources also significantly affected root/shoot ratio of neighboring plant *A. ordosica* in Experiment I, but had no significant effects on it in Experiment II. It may because of different neighboring plant size in two experiments. Larger plant may need greater water gradient and greater ability of hydraulic redistribution to affect plant biomass allocation. The quantitative study on plant hydraulic redistribution ability was necessary.

The horizontal hydraulic redistribution of soil resources not only benefits the donor plants and their neighboring plants and soil microbes (Caldwell et al., 1998) but also may influence plant interactions, community structure and ecosystem processes, such as the cycling of water, carbon, and other nutrients (Horton and Hart, 1998; Leﬄer et al., 2005). Because the distance that resources are redistributed increases with a large, integrated clonal network, the effect of horizontal hydraulic distribution at the community and ecosystem levels can be strong, particularly in drylands in which both hydraulic redistribution and large clonal networks are common. Our results also suggest that plant clonality may have a profound effect at the ecosystem level because clonal plants mediate the redistribution of soil resources. However, the effects of plant clonality in communities and ecosystems require further study (Wilsey, 2002; Yu et al., 2009; Cornelissen et al., 2014), and future studies should be designed to test for these effects in the field.

We expected that an increase in the water gradient between the donor and the recipient ramets of *P. anserina* would increase the translocation of both water and nitrogen and, as a consequence, increase the beneficial effects of clonal integration on *P. anserina* and also possibly on *A. ordosica*. However, with an increase in the water gradient, only the RD in soils increased, and the RN decreased in soils and in *A. ordosica*. High water treatment may also dilute the D concentration in soils under high water treatment, to further decrease RD in soil under high water treatment. Additionally, the increase in the water gradient did not significantly affect the RN or the RD in *P. anserina* or the RD in *A. ordosica* and did not increase the effect of clonal integration on the biomass of *P. anserina* or *A. ordosica.* Possible explanations may be that the low water supply treatment decreased nitrogen availability, and the ability of *A. ordosica* to uptake water and translocate nitrogen were not fully coupled (de Kroon et al., 1998).

Notably, our results were based on a single clone of *P. anserina*, and therefore, the extrapolation of the results to other systems might be limited. In future research, multiple genotypes and also multiple species should be examined for integration-mediated resource distribution. Additionally, we did not calculate water or nitrogen fluxes between plant-soil-plant systems, and to quantify these fluxes, fine-resolution experiments are required. Despite these limitations, our findings confirmed that the long-distance redistribution of resources through integrated clonal networks is possible.

## Conclusion

Clonal integration can mediate the redistribution of resources among different microsites and benefit neighboring plants. Therefore, clonal integration may be a previously undescribed mechanism facilitating plant water and nutrient cycling in ecosystems. In heterogeneous environments, clonal integration may affect the ecosystem at two levels: as a response trait that helps clonal plants perform better in heterogeneous habitats and as an effect trait that modifies habitat heterogeneity and affects interspecific interactions and possibly the community structure and ecosystem processes (Magyar et al., 2004; Cornelissen et al., 2014). We propose that future studies should focus on the effects of plant clonality on communities and ecosystems.

## Author Contributions

MD and X-HY directed and coordinated this study; X-HY, Y-LZ, Z-LL, S-QG, and Y-BS carried out the fieldwork and lab analyses; X-HY, Y-LZ, F-HL, and MD did the data analysis and wrote the first manuscript draft. All authors commented on the manuscript and consent with the submitted version.

## Conflict of Interest Statement

The authors declare that the research was conducted in the absence of any commercial or financial relationships that could be construed as a potential conflict of interest.
