# Supplementary material for: Plant Clonal Integration Mediates the Horizontal Redistribution of Soil Resources, Benefiting Neighboring Plants
Source: Front Plant Sci. 2016 Feb 5;7:77. doi: 10.3389/fpls.2016.00077 (PMC4742616; doi:10.3389/fpls.2016.00077)

Supplementary Information

Plant clonal integration mediates horizontal redistribution of soil resources, benefiting neighbor plants

Xue-Hua Ye[1], Ya-Lin Zhang[1,3], Zhi-Lan Liu[1], Shu-Qin Gao[1], Yao-Bin Song[1, 2], Feng-Hong Liu[1,4] and Ming Dong[1*, 2]

*To whom correspondence should be addressed. E-mail: dongming@ibcas.ac.cn

Supplementary Figure S1 The ratio of $\delta^{15}N$ (RN, above, in Experiment I) and δD (RD, below, in Experiment II) in soil (A, C) and *P. anserina* leaves (B, D) in the labeled, donor containers with the high water supply, which were connected to the *P. anserina* ramets grown with the high, medium and low water supply. All means are significantly bigger than 1 (by one-sample t T tests). Results (F and *P* values) of one-way ANOVA suggest that there was no difference among the three water treatments.

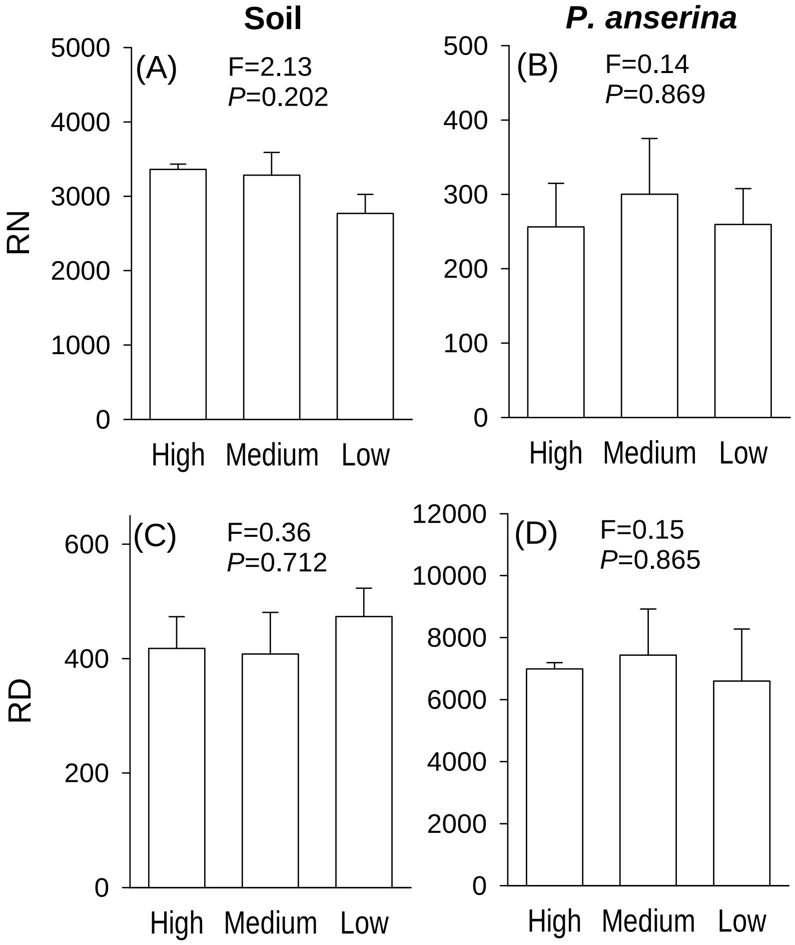

Supplement: Supplementary file 1 [file Image_1.PDF]
